# *Enter* exitrons

**DOI:** 10.1186/s13059-015-0704-3

**Published:** 2015-07-07

**Authors:** Dorothee Staiger, Gordon G. Simpson

**Affiliations:** Molecular Cell Physiology, Faculty of Biology, Bielefeld University, Bielefeld, Germany; Division of Plant Sciences, Life Sciences, University of Dundee at The James Hutton Institute, Invergowrie, DD2 5DA UK; Cell & Molecular Sciences, The James Hutton Institute, Invergowrie, DD2 5DA UK

## Abstract

Exitrons are exon-like introns located within protein-coding exons. Removal or retention of exitrons through alternative splicing increases proteome complexity and thus adds to phenotypic diversity.

## Introduction

Alternative splicing plays a decisive role in shaping the transcriptome [1, 2]. In a recent issue of *Genome Research*, Vienna-based Marquez and colleagues unify a previously disparate set of observations to define a class of alternatively spliced introns that they name ‘exitrons’ (a Viennese mélange of ‘exon’ and ‘introns’) [3]. Exitrons comprise protein-coding intron sequences within protein-coding exons and add a new twist to how protein diversity can arise through alternative splicing.

## Moving on from retained introns

For some time, the most common form of alternative splicing in plants was suggested to be intron retention [4]. The regulatory significance of such events is debated. Transcripts with retained introns tend not to be exported from the nucleus [5, 6]. However, in *Arabidopsis thaliana*, some transcripts with retained introns can be detected on polysomes [4]. A previous study by Marquez and colleagues of alternative splicing in *A. thaliana* then showed that retained intron transcripts constitute a relatively small fraction of all transcript isoforms detected from particular genes [7]. Consequently, they argued that such retained introns likely reflect transcripts still being processed rather than regulatory alternative splicing. The focus on retained introns revealed a subset in protein-coding exons, and it is these that Marquez and colleagues in their latest study now define as exitrons [3].

In a largely computational-based analysis, Marquez and colleagues used their previous *A. thaliana* RNA-Seq data [7] to characterize mRNA exitrons. They broaden the relevance of their findings by identifying exitrons in RNA-Seq data from other plant and animal species and by examining how exitron processing is sensitive to cell type and environmental conditions and can ultimately influence protein function. In this way, they make a compelling case for researchers to focus on, and think differently about, the evolution, regulation and impact of a discrete set of alternative splicing events.

## Distinguishing features of exitrons

Exitrons are defined as introns within protein-coding exons that, when retained, maintain the protein-coding potential of the transcript. Marquez and colleagues argue that four features distinguish exitrons from other introns: high GC content, absence of stop codons, overrepresentation of a size corresponding to multiples of three nucleotides, and prevalence of synonymous substitutions (as usually observed for exonic sequences). Consequently, the fate of transcripts with retained exitrons can also be distinguished from transcripts with retained conventional introns. First, transcripts with retained exitrons are exported from the nucleus to the cytoplasm and translated so that exitron splicing affects protein features, whereas transcripts with retained conventional introns are recognized as incompletely processed and retained in the nucleus, with the result that translation is precluded [6, 8]. Second, only splicing of exitrons with a length not divisible by three has the potential to introduce premature termination codons, whereas retention of conventional introns typically results in premature termination codons. Third, transcripts with retained exitrons are typically the major isoform, whereas transcripts with retained introns are usually of low abundance [5, 7]. Thus, both form and function distinguish exitrons from conventional introns.

Somewhat satisfyingly, Marquez and colleagues simultaneously resolve the debate around *A. thaliana* transcripts with retained introns found on polysomes by revealing that all such previously detected examples are actually unspliced exitrons [3, 4].

## Exitrons are widely conserved

Approximately 1000 exitrons were found in *A. thaliana*, representing 11 % of known intron retention events, and 3.3 % of *A. thaliana* protein-coding genes appear to have exitrons. However, exitrons are not restricted to *A. thaliana*. Marquez and colleagues define examples of exitron splicing not only in other plant species but in metazoan and human cells too. A search of RNA-Seq data obtained from different human tissues and breast cancer samples detected approximately 900 alternatively spliced exitrons, several of which are supported by proteomics data [3]. Some exitron splicing events are conserved between plants and humans. For example, excising an exitron in the *A. thaliana* pre-mRNA encoding eukaryotic translation initiation factor 4A that removes a domain crucial for eIF4A function was found at the very same position in human eIF4A1.

## Impacts of exitrons

Introns with multiples of three nucleotides are rare, whereas exitron sequences are often multiples of three nucleotides, suggesting that they are under similar evolutionary pressure to exons to maintain the reading frame. Consequently, exitron splicing can lead to internally deleted proteins. Exitrons are enriched in sequences coding for posttranslational modifications such as phosphorylation, ubiquitylation, sumoylation, S-nitrosylation and lysine acetylation, and thus exitron splicing can impact the activity or interactions of a protein. Notably, intrinsically disordered regions, rather than defined domains, prevail in protein sequences encoded by exitrons.

Is exitron splicing regulated? As exitrons have relatively weak splice sites and are GC-rich, exitron splicing might be inherently inefficient. However, Marquez and colleagues find that the processing of individual exitrons is differentially affected in different tissues of both humans and plants and is distinctly responsive to particular stress responses in *A. thaliana* and to cancer progression in humans. Thus, alternative splicing of exitrons contributes to transcriptome diversity in specific situations.

## What’s in a name?

Marquez and colleagues were not the first to identify retained introns within protein-coding exons. The peculiarity of such genomic features or splicing events had led others to single them out and refer to them variously as: “intron retention”, “removing an intron from within an exon”, “intra-exonic splicing”, “internal splicing event in exon”, “internal alternative splice sites”, “cryptic introns” or “cryptic splice sites located in exon” [3]. None of these terms is particularly satisfactory, and “cryptic splice sites”, for example, was originally introduced to define splice sites activated upon mutation of authentic splice sites [9]. Most recently, a classification of retained introns in humans based on evolutionary conservation also defined a minor group (type B) of introns located within exons of protein-coding and non-protein-coding RNAs [5].

Marquez and colleagues unify the distinctive properties of such intra-exon intron sequences in different organisms and classify them under a single heading as ‘exitrons’. As a result, previous ambiguity is clarified, providing a shared language for their identification that can improve the accuracy and granularity of genome annotation. For example, almost 20 % of exitrons are found in genes previously annotated as intron-less [3].

This conceptualization of exitrons also triggers focused thinking on how they might be regulated and how they evolve. For example, the influence on co-transcriptional splicing of distinct chromatin modifications and nucleosome positioning associated with exons and introns would be broken in the processing of exitrons. Therefore, in what ways might the processing and regulation of exitron splicing differ from that of conventional introns?

Clearly, exitrons are retained introns. But there is a rationale to move away from this ‘catch all’ classification to reflect a more nuanced understanding. Indeed, this refinement of definitions is not restricted to exitrons: recently, a subset of introns retained in nuclear localized, polyadenylated transcripts were defined as “detained introns” [10]. This terminology derives from insight that reveals that such incompletely spliced transcripts can be rate-limiting intermediates in regulated splicing that impact specific gene expression profiles. In other words, the entry of ‘exitrons’ and ‘detained introns’ into our RNA dictionary reflects the fact that the more general term ‘intron retention’ spans different phenomena that we can now begin to tease apart.
